# Oral iron supplementation after antibiotic exposure induces a deleterious recovery of the gut microbiota

**DOI:** 10.1186/s12866-021-02320-0

**Published:** 2021-09-28

**Authors:** Thibault Cuisiniere, Annie Calvé, Gabriela Fragoso, Manon Oliero, Roy Hajjar, Emmanuel Gonzalez, Manuela M. Santos

**Affiliations:** 1 Nutrition and Microbiome Laboratory, Institut du cancer de Montréal, Centre de recherche du Centre hospitalier de l’Université de Montréal (CRCHUM), CRCHUM - R10.426, 900 rue Saint-Denis, Montréal, Québec H2X 0A9 Canada; 2 Digestive Surgery Service, Centre hospitalier de l’Université de Montréal, Montréal, Canada; 3 Canadian Centre for Computational Genomics, Department of Human Genetics; and Microbiome Platform Research, McGill Interdisciplinary Initiative in Infection and Immunity, McGill University, Montréal, Canada; 4 Department of Medicine, Faculty of Medicine, Université de Montréal, Montréal, Canada

**Keywords:** Iron, Antibiotics, Gut microbiota, Dysbiosis, Anemia, Inflammation

## Abstract

**Background:**

Oral iron supplementation is commonly prescribed for anemia and may play an important role in the gut microbiota recovery of anemic individuals who received antibiotic treatment. This study aims to investigate the effects of iron supplementation on gut microbiota recovery after antibiotics exposure.

**Results:**

Mice were subjected to oral antibiotic treatment with neomycin and metronidazole and were fed diets with different concentrations of iron. The composition of the gut microbiota was followed throughout treatment by 16S rRNA sequencing of DNA extracted from fecal samples. Gut microbiota functions were inferred using PICRUSt2, and short-chain fatty acid concentration in fecal samples was assessed by liquid-chromatography mass spectrometry. Iron supplementation after antibiotic exposure shifted the gut microbiota composition towards a Bacteroidetes phylum-dominant composition. At the genus level, the iron-supplemented diet induced an increase in the abundance of *Parasutterella* and *Bacteroides*, and a decrease of *Bilophila* and *Akkermansia*. *Parasutterella excrementihominis*, *Bacteroides vulgatus*, and *Alistipes finegoldii*, were more abundant with the iron excess diet. Iron-induced shifts in microbiota composition were accompanied by functional modifications, including an enhancement of the biosynthesis of primary bile acids, nitrogen metabolism, cyanoamino acid metabolism and pentose phosphate pathways. Recovery after antibiotic treatment increased propionate levels independent of luminal iron levels, whereas butyrate levels were diminished by excess iron.

**Conclusions:**

Oral iron supplementation after antibiotic therapy in mice may lead to deleterious changes in the recovery of the gut microbiota. Our results have implications on the use of oral iron supplementation after antibiotic exposure and justify further studies on alternative treatments for anemia in these settings.

**Supplementary Information:**

The online version contains supplementary material available at 10.1186/s12866-021-02320-0.

## Background

The gut microbiota is the population of microorganisms inhabiting the gastrointestinal tract. Crosstalk between the host and gut microbiota is important for several key functions in the health of the host, such as immunity maintenance, nutrient metabolism and protection against pathogens [1]. Perturbations in the gut microbiota may lead to disturbed microbial homeostasis, a state termed dysbiosis. Antibiotic treatments are among the strongest inducers of gut dysbiosis [2, 3]. Antibiotics are routinely administered to treat active infections or for prophylaxis prior to invasive endoscopic or surgical interventions [4]. Antibiotic-induced dysbiosis leads to a marked reduction in alpha-diversity [5, 6] and beneficial bacteria [6], a decrease in the levels of beneficial short-chain fatty acids (SCFAs) [7], a weakening of the gut barrier function, and inflammasome activation [7, 8]. Most importantly, antibiotic-induced dysbiosis is linked to an increased abundance of enterobacteria species [7], including pathogenic species [9]. Once antibiotic treatments are stopped, the gut microbiota can return to its initial composition [10]. However, this return to baseline or recovery may be impaired by several exogenous factors such as nutrient availability in the gastrointestinal tract [11].

Iron availability may constitute a determinant factor in microbiota recovery because iron functions as a co-factor in iron-containing proteins in redox reactions, electron transport chain mechanisms, and metabolic pathways, underscoring its role in the growth and replication of most microorganisms [12]. As iron is a limiting growth factor for most bacteria [13], the host has a highly regulated system to control levels of free iron within the gut lumen [14], resulting in intense competition between host cells and gut bacteria for iron acquisition [15, 16]. However, oral iron supplementation increases the iron availability in the gut as most of the supplemented iron is not absorbed [17, 18]. Excess iron in the gut lumen may potentially activate latent virulence genes in commensal bacteria, turning them into pathobionts [19]. Hence, the host iron status and dietary iron availability have a profound impact on microbial gut communities [20]. Importantly, iron supplementation has also been shown to favor the growth of pathogenic gut bacteria and the occurrence of intestinal injury [21]. The adverse effects of iron may be exacerbated in dysbiotic settings, with unabsorbed luminal iron from diet or iron supplementation further fueling the growth and virulence of gut bacterial pathogens. In turn, iron-mediated modification of the gut microbiome, particularly during the recovery phase after antibiotic therapy, may have profound consequences for the health of the host.

Oral iron supplementation, mostly in the form of ferrous sulphate [22], is a universal treatment for anemia [23]. Both antibiotic treatment and iron supplementation are administered to anemic patients needing gastrointestinal surgery due to digestive diseases [24, 25]. In these cases, antibiotic treatment is given as a prophylaxis before surgical interventions to prevent infections and consists routinely of oral regimens of neomycin and metronidazole [26, 27], which confer a broad coverage against gastrointestinal bacteria.

In this study, we evaluated the effects of oral iron supplementation on the gut microbiota during the crucial period of recovery after exposure to antibiotics.

## Results

### The structure of gut microbiota communities is affected by dietary iron supplementation after antibiotic exposure

**Fig. 1:**
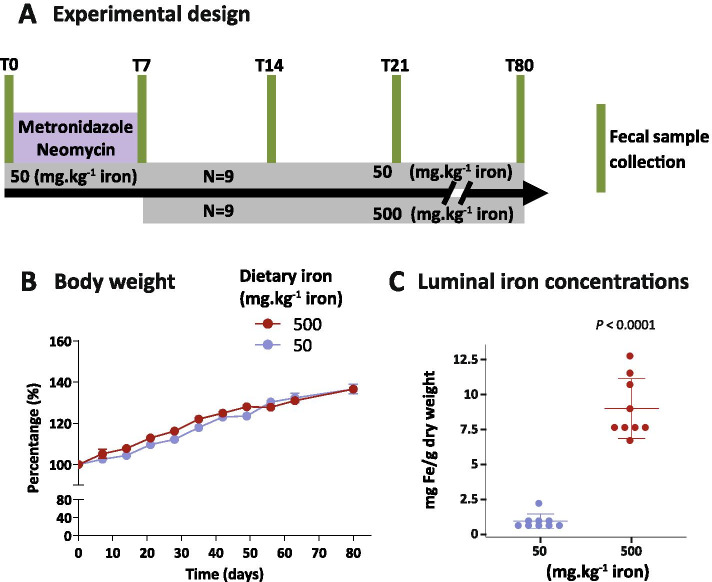
Dietary iron supplementation increases luminal iron concentration of the gut without affecting body weight. **A** Schematic representation of the experimental design starting with mice fed a diet sufficient in iron and antibiotic delivery for one week, followed by either an iron-sufficient (50 mg.kg^-1^) or iron-supplemented (500 mg.kg^-1^) diet until day 80 (T80). **B** Body weight (means ± SEM) between iron-sufficient and iron-supplemented groups. **C** Fecal non-heme iron measured by ferrozine assay at T80 (means ± SD). *N* = 9 per group. Differences were assessed by multiple *t*-test corrected for false discovery rate (*fdr*) (B) and Welch *t*-test (C)

To assess gut microbiota recovery from antibiotic treatment in the presence or absence of oral iron supplementation, mice were first treated with metronidazole and neomycin for one week while fed an iron-sufficient diet. Next, mice were divided into two groups: one continued to be fed the iron-sufficient diet while the other was switched to an iron-supplemented diet (Fig. 1A).

During the experimental period, mice gained weight and no differences were detected between the iron-sufficient and iron-excess groups (Fig. 1B). To confirm an increase in luminal iron concentration in mice kept on the high iron diet, we measured iron levels in fecal samples at day 80 (T80, Fig. 1C). The iron-supplemented diet induced a 10-fold increase in luminal iron concentration.

We used 16S rRNA amplicon sequencing to profile the fecal microbiota of mice before (T0) and after (T7) antibiotic exposure as well as during the recovery period (T14, T21 and T80). Raw data with the results of the 16S rRNA gene microbial profiling analysis including complete taxonomic profiles, alpha-diversity, and inferred functional profiles are presented in Additional file 1

**Fig. 2:**
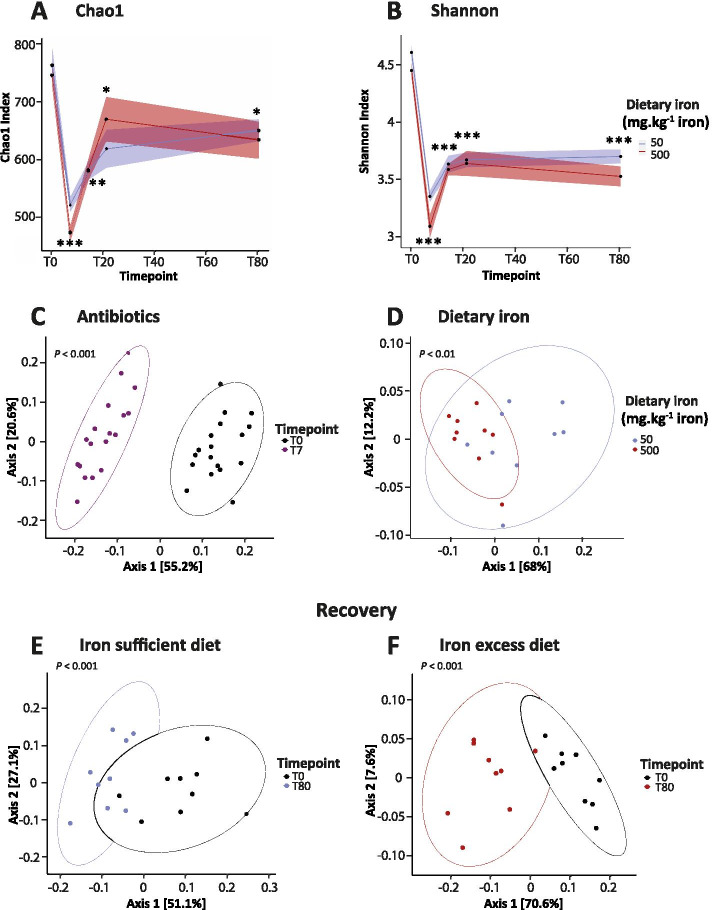
Recovery of gut microbiota composition is incomplete at T80 and affected by luminal iron concentration. **A** Chao1 and **B** Shannon measurements of alpha-diversity. Ribbons represent the standard error of the mean (SEM). **C**-**F** Principal coordinates analysis (PCoA) of weighted UniFrac distance matrix. *N* = 18 mice per group for antibiotic exposure and *N* = 9 mice per group for iron diets. Differences were assessed by Welch *t*-test (**A**, **B**) and Adonis (**C**-**F**). * *P* < 0.05, ** *P* < 0.01, *** *P* < 0.0001, comparison between T0 and T7 (*N* = 18), T14, T21 and T80 (*N* = 9)

To assess the effects of dietary iron on richness and evenness of the gut microbiota during recovery, alpha-diversity Chao1 and Shannon indexes were computed. Analysis of alpha-diversity indexes revealed that antibiotics induced a strong perturbation characterized by a significant decrease of Chao1 (755 ± 16 T0 *vs* 497 ± 11 T7, *P* < 0.00001) and Shannon indexes (4.5 ± 0.05 T0 *vs* 3.2 ± 0.06 T7, *P* < 0.00001) (Fig. 2A-B). As reported by others [28], our results confirm that antibiotic exposure induces a strong decrease in alpha-diversity, affecting both richness and evenness of the gut microbiota. To assess how iron supplementation affects the recovery of alpha-diversity, we compared baseline (T0) with the last time point (T80) in both groups. As shown in Fig. 2A-B, Chao1 and Shannon indexes at T80 remained significantly lower compared to T0, independent of dietary iron consumption (iron-sufficient diet: Chao1 650 ± 19 T80, *P* < 0.05; Shannon 3.7 ± 0.09 T80, *P* < 0.00005; and iron excess diet: Chao1 634 ± 32 T80, *P* < 0.05; Shannon 3.5 ± 0.09 T80, *P* < 0.00001). No difference in alpha-diversity was detected between mice fed the iron-sufficient and iron-supplemented diets at T80.

Next, analysis of the level of differences between experimental groups (beta-diversity) was computed by weighted UniFrac and their principal coordinate analysis (PCoA). Analysis of antibiotic-induced dissimilarity revealed that axis 1 captured most of the variance among microbiota samples (55.2%) depicting drastic changes from baseline in antibiotic-treated gut microbiota structure (*P* < 0.001) (Fig. 2C).

The effects of iron were then assessed by comparing both groups at the last timepoint (T80). The recovery of community structures was significantly affected under different luminal iron concentrations (Fig. 2D) as shown by the 68% variation captured by PCoA axis 1 (*P* <0.01). Furthermore, a clear pattern significantly differentiating the gut microbiota at baseline compared to the end of the experiment (T80) was found independent of dietary iron levels (Fig. 2E-F) (PCoA axis 1: iron-sufficient diet 51.1%, *P* < 0.001 and iron excess diet 70.6%, *P* < 0.001).

Taken together, these results indicate that antibiotic exposure reduced alpha-diversity and differentially clustered the structure of the gut microbiota. Dietary iron affected gut microbiota structure at the end of the recovery phase and did not affect alpha-diversity. Thus, after antibiotic exposure, iron impacted the microbiota composition without affecting alpha-diversity.

### Iron supplementation after antibiotic exposure influences the gut microbiota composition at the phylum, family, genus, and species levels

**Fig. 3:**
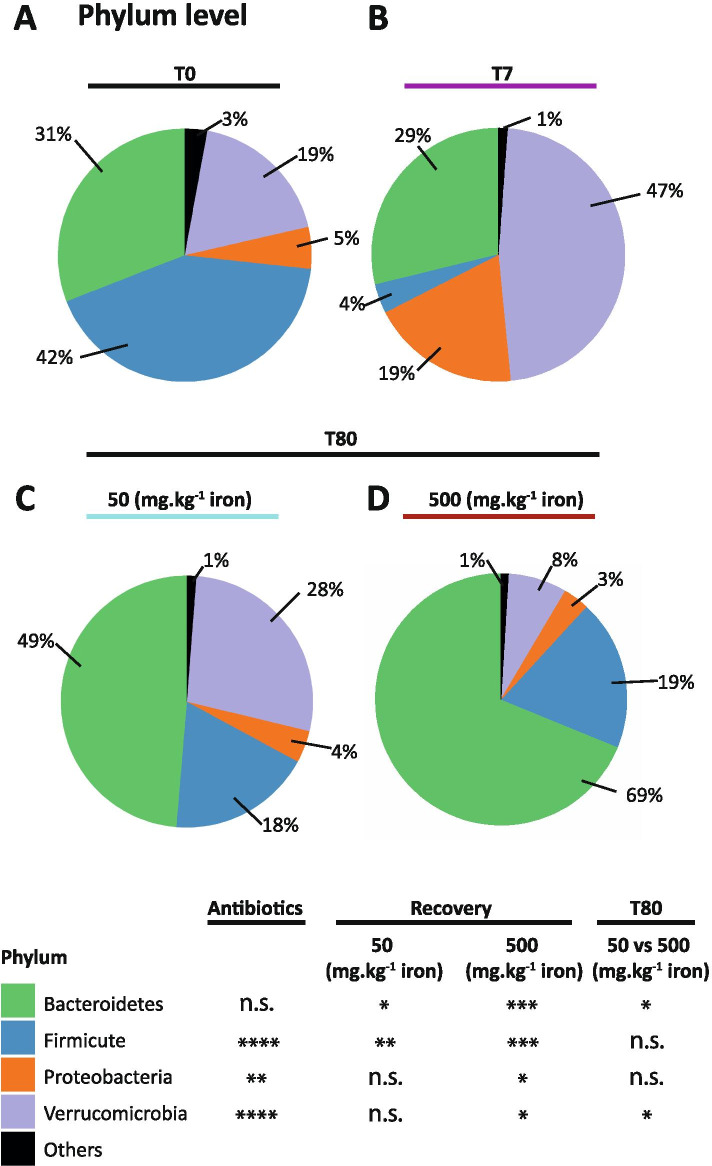
Antibiotics induce major perturbations in relative abundances and iron shapes the gut microbiota during recovery. Pie charts of phyla relative abundances at (**A**) T0 (*N* = 18), (**B**) T7 (*N* = 18) and T80 for mice fed (**C**) iron-sufficient diet (*N* = 9) and (**D**) iron excess diet (*N* = 9). Differences were assessed by Welch *t*-test; n.s., non-significant, * *P* < 0.05, ** *P* < 0.01, *** *P* < 0.001, **** *P* < 0.0001

**Fig. 4:**
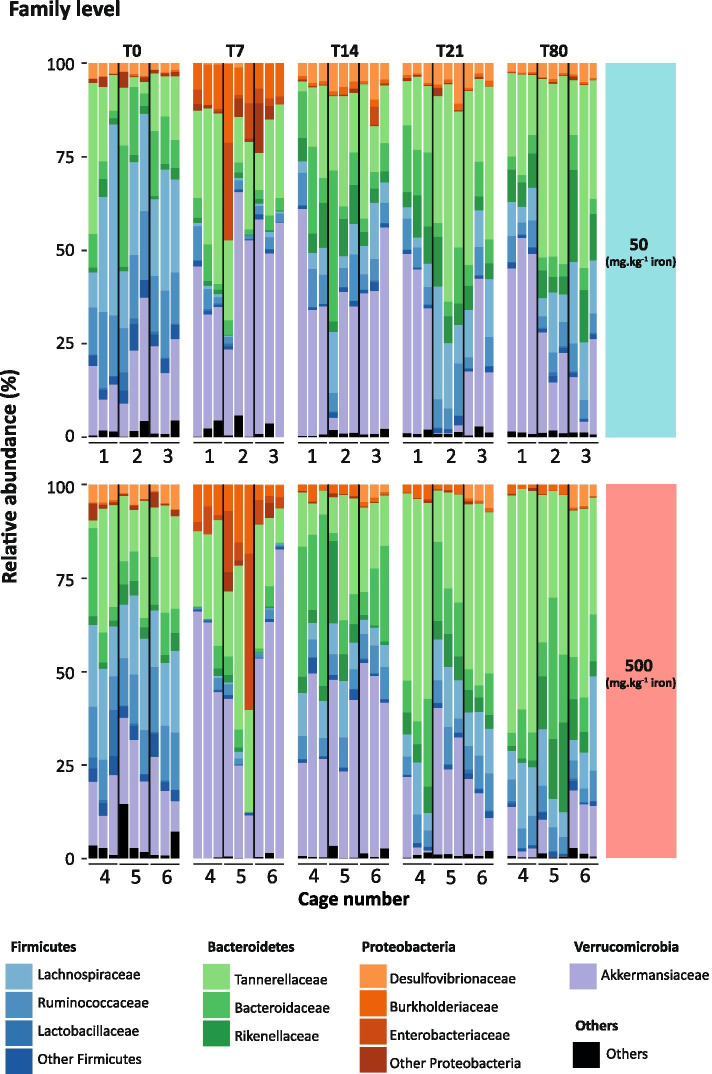
Oral iron supplementation shapes gut microbiota recovery from antibiotic exposure at family level. Bar graph of relative abundances of families throughout the experiment. Each stacked bar represents a single mouse and the vertical lines demark cages. Families representing more than 5% of the relative abundance are shown

To detect perturbations in the gut microbiota composition after antibiotic exposure, bacterial relative abundance at the phylum and family levels were analyzed (Figs. 3 and 4). Antibiotic exposure induced a major perturbation of the relative abundances of whole phyla (Fig. 3A-B). In particular, antibiotics induced a bloom of Verrucomicrobia (19% T0 *vs* 47% T7, *P* < 0.00005) driven by Akkermansiaceae, and a rise in Proteobacteria (5% T0 *vs* 19% T7, *P* <0.002) driven mostly by Enterobacteriaceae and other Proteobacteria, while all other phyla decreased in terms of relative abundance. Most importantly, the relative abundance of Firmicutes was strongly diminished by antibiotic treatment (42% T0 *vs* 4% T7 *P* < 0.00001). Taken together, these results indicate that antibiotics shift gut microbiota communities towards an unbalanced state.

Analysis of the relative abundance comparing T0 *vs* T80 (recovery) revealed persistent modifications at the phylum level in mice fed iron-sufficient and iron-supplemented diets (Fig. 3C-D). Independent of dietary iron intake, Firmicutes did not return to basal levels as shown in Fig. 3A (iron sufficient diet: 42% T0 *vs* 18% T80, *P* < 0.005; and iron excess diet: 19% T80, *P* < 0.0005). In contrast, Bacteroidetes were more abundant at T80 in both groups but at a higher extent in mice fed the iron-supplemented diet (iron-sufficient diet: 31% T0 *vs* 49% T80, *P* < 0.05; and iron excess diet: 69% T80, *P* < 0.0005), indicating that the Bacteroidetes expansion after antibiotic treatment was dependent on luminal iron availability. In addition, iron supplementation strongly suppressed Verrucomicrobia expansion triggered by antibiotic treatment (iron-sufficient diet, 28% T80 *vs* iron excess diet, 8% T80, *P* < 0.05). At T80 follow-up, the gut microbiota composition did not return to baseline, and the iron supplementation induced a shift towards Bacteroidetes domination mostly belonging to the Tannerellaceae family (iron-sufficient diet: 18% T0 *vs* 34% T80, *P* = 0.07; and iron excess diet: 46% T80, *P* < 0.01; Figs. 3 and 4).

**Fig. 5:**
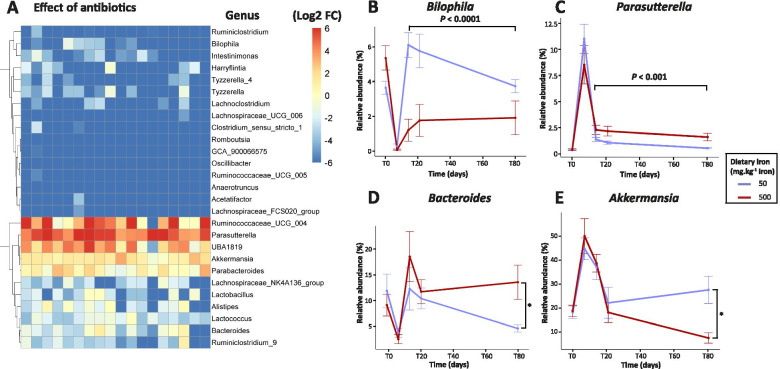
Oral iron supplementation shapes the gut microbiota recovery from antibiotic exposure at the genus level. **A** Heatmap of log2 fold change (FC) of the relative abundances of the significantly different genera between mice before (T0) and after (T7) antibiotic exposure (GAMLSS-BEZI analysis). Each square represents one mouse (*N* = 18 per group). **B**-**C** The effect of iron supplementation on the relative abundance of genera that were significantly affected through the full recovery phase (T14, T21 and T80). **D**-**E** The effect of iron supplementation on the relative abundance of genera that remained significantly affected at the last time point (T80). Vertical bars represent the standard error of the mean (SEM) (*N* = 9 per group)

To detect differentially abundant bacterial genera and species induced by the iron-enriched diet, we applied Generalized Additive Models for Location, Scale and Shape (GAMLSS) with a zero-inflated beta (BEZI) family (GAMLSS-BEZI). Antibiotic treatment (T0 *vs* T7) resulted in a significant decrease of *Bacteroides* and *Alistipes,* and an increase of *Parabacteroides* genera from the Bacteroidetes phylum (Fig. 5A and Additional file 2, Table 1). In addition, 19 genera in the Firmicutes phylum had significantly decreased, while the relative abundance of two genera, namely *Ruminococcaceae UCG 004* and *UBA1819*, increased. Two Proteobacteria genera had also significantly changed, with a reduction of *Bilophila* and a relative expansion of *Parasutterella*. Finally, antibiotic exposure also significantly increased the relative abundance of *Akkermansia* (Verrucromicrobia phylum). The relative abundance of genera that remained affected until the last time point (T80) compared to baseline (T0) are presented in Additional file 2, Table 2 (iron-sufficient diet, 50 ppm iron) and Table 3 (iron excess diet, 500 ppm iron).

Most importantly, dietary iron levels affected the return to basal levels through the recovery period (T14, T21 and T80) of two genera. *Bilophila* showed a diminished recovery rate in mice fed the iron excess diet (Fig. 5B, *P* < 0.0001), and *Parasutterella* presented a higher relative abundance in mice consuming the iron excess diet (Fig. 5C, *P* < 0.001). The relative abundance of two additional genera remained significantly altered only at the last time point (T80). *Bacteroides* was increased in mice fed the iron excess diet (Fig. 5D; *P <* 0.05) and *Akkermansia* was reduced, with iron supplementation suppressing its expansion (Fig. 5E; *P <* 0.05)

**Fig. 6:**
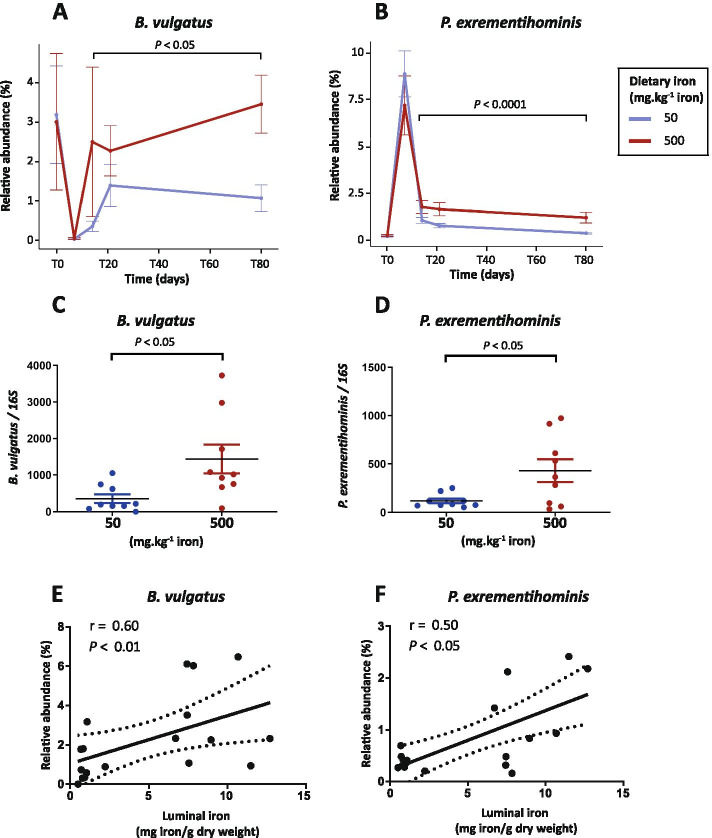
Relative abundance of bacterial species in iron-supplemented mice after antibiotic exposure. **A**-**B** Relative abundance of the two bacterial species that were detected as significantly different in abundance by GAMLSS-BEZI and **C**-**D** validated by real-time PCR, in mice fed an iron excess diet compared to mice fed an iron-sufficient diet during the recovery phase. Differences were assessed by Welch *t*-test. Bars represent the standard error of the mean (SEM) (*N* = 9 per time point and group). **E**, **F** Spearman’s correlations between luminal iron concentration at T80 of (**E**) *B. vulgatus* and (**F**) *P. excrementihominis*. r represents the correlation coefficient and dotted lines represent confidence interval at 95%. (*N* = 18)

We further identified two species, namely *Bacteroides vulgatus* (Bacteroidetes) and *Parasutterella excrementihominis* (Proteobacteria), which were differentially abundant during the recovery phase in mice fed with iron-supplemented diet compared to the control iron-sufficient diet (Fig. 6). *B. vulgatus* (Fig. 6A) was more sensitive to antibiotic exposure, with a relative abundance of 3.09% ± 1.03 at T0 and 0.04% ± 0.015 at T7 (*P* < 0.0001). The iron-supplemented diet induced a fast return to baseline of the relative abundance of *B. vulgatus* at T14, which then remained stable during the recovery phase. In contrast, the iron-sufficient diet induced a significantly lower relative abundance at all remaining timepoints.

*P. excrementihominis* (Fig. 6B) exhibited resistance to antibiotic exposure as shown by a 34-fold increase in its relative abundance (0.24% ± 0.04 at T0 *vs* 8.04% ± 0.99 at T7, *P* < 0.0001). Oral iron supplementation during the recovery phase induced a higher relative abundance of *P. excrementihominis* compared to the iron-sufficient diet. Notably, *P. excrementihominis* remained higher at T80 compared to T0 (iron-sufficient diet: *P* < 0.002, iron excess diet: *P* < 0.006), independent of dietary iron during the recovery phase.

To validate the results obtained from 16S rRNA analysis at the species level, we performed real-time PCR analysis on samples obtained at the last time point, T80. As shown in Fig. 6C-D, real-time PCR analysis confirmed the expansion of *B. vulgatus* and *P. excrementihominis* in the gut microbiota of mice fed the iron excess diet.

The data so far indicate that these two bacterial species preferentially grew in an iron-rich luminal environment during recovery from antibiotic exposure. To further analyze the dependency of their growth on iron luminal concentrations, we applied Spearman’s correlations analysis (Fig. 6E-F) and showed that luminal iron concentrations significantly correlated with the relative abundance of *B. vulgatus* (*r =* 0.60, *P* < 0.01) and *P. excrementihominis* (*r =* 0.50, *P* < 0.05) measured at T80.

GAMLSS-BEZI analysis revealed a third species, *Alistipes finegoldii* (Bacteroidetes) (Additional file 2, Fig. 1A) that exhibited a significant decline by a factor eight of its relative abundance after antibiotic exposure (0.41% ± 0.079 at T0 *vs* 0.053% ± 0.018 at T7, *P* < 0.0001). The relative abundance of *A. finegoldii* remained lower in mice fed the iron-sufficient diet compared to mice fed the high iron diet throughout the recovery phase and was higher at the endpoint (T80) for both groups compared to baseline. Further real-time PCR confirmed that at T21, but not at T80, *A. finegoldii* was present at higher levels in mice fed the iron excess diet compared to mice kept on the iron-sufficient diet (Additional file 2, Fig. 1B-C).

### Iron supplementation during gut microbiota recovery after antibiotic exposure induces changes in inferred gut microbiota functions

**Fig. 7:**
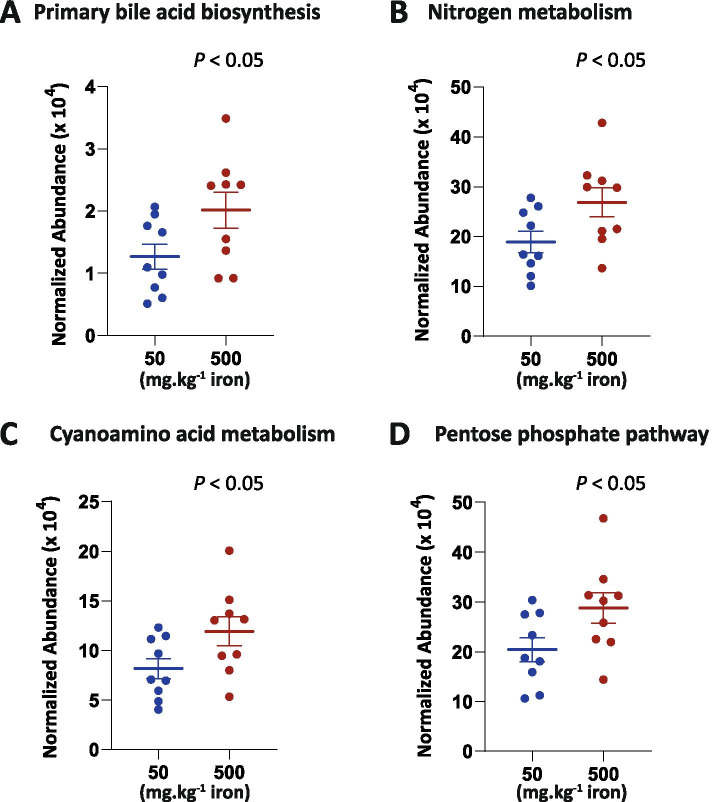
Dietary iron during recovery from antibiotic exposure significantly affects gut microbiota functions. Normalized relative abundances of Kyoto Encyclopedia of Genes and Genomes (KEGG): **A** primary bile acid biosynthesis, **B** nitrogen metabolism, **C** cyanoamino acid metabolism, and **D** pentose phosphate pathways were detected as significantly different by White’s *t*-test between mice under iron-sufficient and iron excess diet at T80. Bars represent the standard error of the mean (SEM). (*N* = 9)

PICRUSt2 was used to infer differences in gut microbiota composition to differences in gut microbiota functions. Four differentially abundant functions were detected as significantly more abundant at T80 in mice fed the high iron diet compared with those fed the iron-sufficient diet (Fig. 7): primary bile acid biosynthesis, nitrogen metabolism, cyanoamino acid metabolism, and pentose phosphate pathway. Microbiota shifts induced by iron were accompanied by a significant increase in the abundance of genes of these four metabolic pathways.

**Fig. 8:**
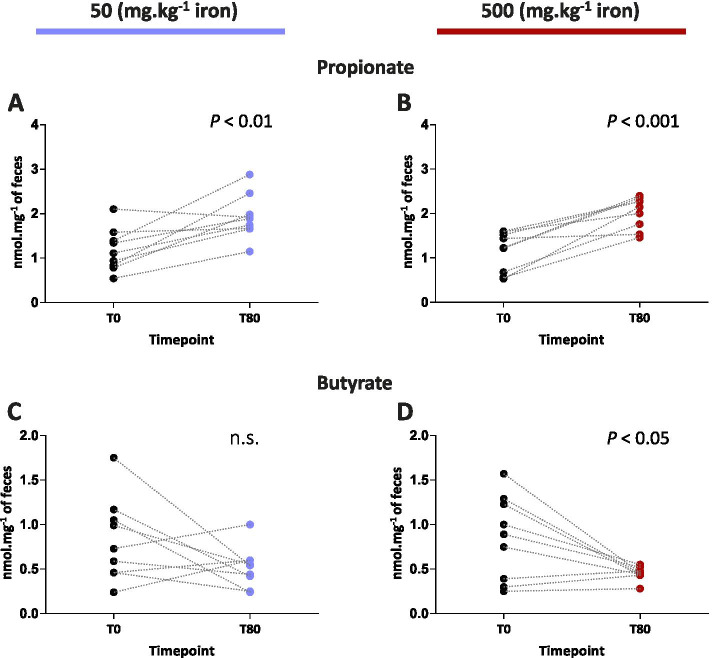
Dietary iron significantly affects fecal SCFAs concentrations. **A**, **B** Comparison of concentrations of fecal propionate and **C**, **D** fecal butyrate in mice fed an iron-sufficient diet (**A** and **C**) and iron excess diet (**B** and **D**). Statistical significances were determined by paired *t-*test. (*N* = 9)

Since the composition of the gut microbiota did not recover to its baseline level at T80 and dietary iron effectively shaped the recovery towards a Bacteroidetes-dominant phylum composition (mainly propionate producers) [29] to the detriment of Firmicutes (mainly butyrate producers) [30], we reasoned that the production of SCFAs was affected. SCFAs are the major fermentation products of the gut microbiota and are involved in diverse biological functions that affect the host’s physiology [31]. We investigated whether fecal SCFAs concentrations at the final timepoint (T80) were affected by the iron-induced recovery from antibiotic exposure (Fig. 8). The concentration of fecal propionate at T80 was significantly increased in mice fed the iron-sufficient and iron-supplemented diets when compared to baseline (T0). Conversely, fecal concentrations of butyrate significantly decreased in mice under the iron-supplemented diet, but not in mice under the iron-sufficient diet. Overall, these results indicate that propionate levels were still above basal levels for more than two months after antibiotic exposure and that iron supplementation significantly prevented the return to baseline of fecal butyrate concentrations.

## Discussion

The aim of this study was to investigate the effect of oral iron supplementation on gut microbiota reconstitution after antibiotic exposure. Several studies have highlighted the recovery phase as a critical component in the return to a balanced state of the gut microbiota [32, 33]. However, the impact of oral iron supplementation in this context has not yet been explored. Antibiotic exposure and anemia are prevalent in the general population; the daily dose of antibiotics per 1000 inhabitants ranges from 4 to 64 worldwide [34], while anemia is present in more than 30% of the population [23] with many requiring iron supplementation [35].

Here, we show that antibiotics disrupted the mouse gut microbiota by dramatically reducing alpha-diversity and inducing differentially clustered microbiota composition. This dysbiotic state was characterized by a dramatic decrease of Firmicutes and a bloom in Verrucomicrobia, Bacteroidetes, and Proteobacteria, which is in accordance with previous studies on the effects of antibiotics on the gut microbiota [36–38]. The gut microbiota composition remained altered until the end of the experiment, as assessed by alpha- and beta-diversity analyses. Moreover, relative abundances at the phylum level were dominated by Bacteroidetes, which includes both beneficial and harmful genera [39]. Interestingly, Bacteroidetes dominance was more pronounced in mice fed the high iron diet to the detriment of the phylum Verrucomicrobia, while Firmicutes remained lower in comparison to baseline, independent of dietary iron. Previous studies suggest that low Firmicutes/Bacteroidetes ratios represent a marker of dysbiosis [40] and report low ratios in overweight and obese individuals [41], and in colon neoplastic lesions [40]. In our study, the Bacteroidetes dominance state was characterized mainly by an increase in the Tannerellaceae family. The high abundance of the Verrucomicrobia phylum at T0 could be explained by the effect of low fiber concentration [42] in our control diet, which began one week prior to antibiotic delivery. In addition, antibiotic exposure is also known to induce an increase in the relative abundance of Verrucomicrobia [28, 36].

At the genus level, we found a decrease of *Bilophila* in iron-supplemented mice after antibiotic treatment. Similarly, previous reports in mice receiving iron supplementation without antibiotic treatment reported a reduction in *Bilophila* [43]. While this genus contains known pathobionts, it has more recently been associated with beneficial effects in neurodegenerative diseases [44] and cardiovascular health [45].

An increase in the genus *Parasutterella* was also found in mice receiving the iron excess diet. *Parasutterella* has been defined as a core component of the gut microbiota and has been correlated with various health outcomes, such as inflammatory bowel disease [46], obesity, and fatty liver disease [47, 48]. In our study, the relative abundance of *P. excrementihominis* increased in mice receiving the iron excess diet after antibiotic exposure. *P. excrementihominis* is a gram-negative, non-spore forming, strict anaerobe that has been associated with the development and progression of irritable bowel syndrome. Taken together, our data suggest that iron supplementation leads to an increased abundance of this genus, promoting gut inflammation, chronic debilitating symptoms and a weakening of the mucosal barrier.

*Bacteroides* is one of the most prevalent genera in the gut microbiota [49], implying that alterations in its abundance may have major effects on gut homeostasis and the host’s health. The increased abundance of *Bacteroides* have been reported to induce different infections in the gynecological, articular, cardiac and neurological systems [50]. This genus is frequently involved as causal agents in intra-abdominal infections and associated bacteremia [50, 51], suggesting a pathogenic potential of *Bacteroides* species, particularly when they escape the gut environment [50]. Our findings are in agreement with previous data reporting an increase in the relative abundance of *Bacteroides* after iron supplementation [52]. In addition, we found a member of this genus, *B. vulgatus*, that was significantly increased in mice receiving iron supplementation. *B. vulgatus* is an anaerobic, gram-positive commensal bacterium, which relies on stealing siderophores produced by other organisms to ensure iron acquisition in the gut [53]. Siderophores are small peptides secreted by bacteria to capture iron from the environment. These siderophore-iron molecules are taken up by bacteria expressing specific siderophore receptors to acquire iron. As a “cheater” species [54], *B. vulgatus* avoids expending metabolic resources to produce siderophores by using the ones produced by other bacterial species. In a gut environment rich in iron as in our iron-supplemented experiments, *B. vulgatus* is likely to benefit with enhanced virulence. This bacterium is one of the most abundant in the gut [55] and has been associated with inflammation [50, 56] via the activation of inflammatory pathways [55]. An increased abundance of *B. vulgatus* in an iron-rich environment is therefore believed to sustain a pro-inflammatory environment, which may in turn alter the gut barrier function and impact the overall health of the host.

Iron supplementation after antibiotic treatment also suppressed the expansion of the *Akkermansia* genus (Verrucromicrobia). Consistent with our results, *Akkermansia* were reported to bloom after antibiotic exposure [57, 58]. This genus has been shown to harbor a protective role against obesity and metabolic syndrome [59]. The most known species in this genus is *Akkermansia muciniphila*, which is associated with benefits that extend beyond metabolic functions and can further promote intestinal homeostasis and healing [60], cognitive functions [61], and the antineoplastic effects of immunotherapy in murine models [62].

At the species level, *A. finegoldii* (phylum Bacteroidetes) was increased, particularly during the recovery of iron-supplemented mice. This bacterium is a gram-negative, strict anaerobe. A higher abundance of the *Alistipes* genus has been associated with ileal inflammation in a mouse model of spontaneous inflammation of the ileum [63]. Oral gavage with *A. finegoldii* induced intestinal inflammation in wild-type, *Il10*^*-/-*^, and lipocalin 2 knockout (*Lcn2*^-/-^) mice [64]. Lcn2 is an antimicrobial protein secreted by the host that sequesters siderophores [65], limiting iron availability [66]. In *Il10*^-/-^ mice, Lcn2 deficiency (*Lcn2*^*-/-/*^*Il10*^*-/-*^ mice) created a niche for the expansion of facultative pathogenic *A. finegoldii* spp., preferentially colonizing the cecum and proximal colon [64]. When transferred into *Il10*^*–/–*^ mice, *A. finegoldii* spp. were sufficient to induce colitis and right-sided tumors, and the authors demonstrated that Lcn2 impacted the *in vitro* growth of *Alistipes* spp. by limiting iron availability [64]. These findings suggest that *A. finegoldii* thrives in an iron-rich environment by a mechanism linked to siderophore-mediated iron uptake.

Differences in gut microbiota composition were accompanied by significant modifications in gut microbiota functions. Oral iron supplementation induced a higher number of genes involved in primary bile acid production by gut bacteria. We also found that genes involved in nitrogen metabolism pathways were present at higher levels in the gut microbiota of mice fed the high iron diet. Bacterial nitrogen metabolism leads to production of ammonia, which is cytotoxic for the colonic epithelium. Expansion of *Bacteroides* and *Akkermansia* have been associated with an increase in fecal ammonia concentrations [67, 68], while a decrease of *Parasutterella* with a decrease of nitrogen metabolism [69]. The pentose phosphate pathway genes were also present at higher levels in mice fed the high-iron diet. An end product of the pentose phosphate pathway is nicotinamide adenine dinucleotide phosphate (NADPH), which has been shown to be protective against oxidative stress [70]. Since iron is a known oxidative stress inducer [71], excess luminal iron may exert selective pressure resulting in a survival benefit to bacteria that can efficiently control oxidative stress.

Finally, bacteria belonging to the Bacteroidetes phylum are the main producers of propionate while Firmicutes bacteria are the main producers of butyrate [30]. Consistently, fecal propionate concentrations increased alongside the abundance of the Bacteroidetes phylum and fecal butyrate concentrations decreased alongside that of the Firmicutes phylum only in mice under the high iron diet. A possible explanation could be the significantly higher relative abundance of the butyrate producer *A. muciniphila* in mice under iron-sufficient diet compared to mice under iron excess diet [72]. Nonetheless, the lack of return to baseline of fecal butyrate concentrations in mice fed the iron-supplemented diet could potentially lead to harmful effects on the gut homeostasis of the host.

## Conclusions

In conclusion, we describe the changes to the gut microbiota after exposure to antibiotics and the effects of oral iron supplementation on its recovery. We also highlighted potential risks associated with long-term disturbances of gut microbiota functions and composition. We identified preferential growth of three species, one Proteobacteria and two Bacteroidetes. Taken together, our results suggest deleterious changes in the gut microbiota after exposure to antibiotics and oral iron supplementation. These findings require further studies to explore their biological effects in animal models and confirm the deleterious impact of oral iron supplementation after antibiotic exposure on intestinal health.

## Methods

### Animal experiments

Four-week old female C57Bl/6 mice were purchased from Charles River Laboratories (Saint-Constant, QC, Canada). Mice were kept under controlled specific pathogen-free (SPF) conditions in the CRCHUM animal facility at a temperature of 22°C, 45-60% humidity and a light-dark cycle of 12-12. They were housed at three mice per cage with *ad libitum* access to chow and water. Cages were enriched with nesting material and changed every two weeks. Mice were allowed one week of acclimation following arrival to the CRCHUM animal facility, and were then switched from standard chow (Teklad TD.2918; Envigo, IN, USA) to an iron-sufficient diet containing 50 mg.kg^-1^ of iron sulphate (Teklad TD.120515; Envigo, IN, USA). On the seventh day of dietary intervention, oral antibiotics including metronidazole (1 mg.ml^-1^, Hospira, St-Laurent, QC, Canada) and neomycin (1 mg.ml^-1^, Sigma, St-Louis, MO, USA) were added to the drinking water for one week. After antibiotic exposure, mice were randomly assigned to two groups and maintained under the iron-sufficient diet or switched to an iron-supplemented diet containing 500 mg.kg^-1^ of iron sulphate (Teklad TD.120517; Envigo, IN, United States) until the end of the experiment. Fecal samples were collected before (T0) and after antibiotic treatment (T7) and at days 14, 21, and 80 (T14, T21, T80), snap-frozen and stored at -80°C. Mice were euthanized using CO_2_ followed by cervical dislocation.

### Iron measurements

Measurement of iron concentration was performed on harvested stool samples by the ferrozine method using the QuantiChrom™ Iron Assay Kit (BioAssay Systems, CA, USA) according to the manufacturer’s protocol and as previously reported [73].

### 16S rRNA sequencing

Bacterial DNA was extracted using Qiagen DNeasy PowerSoil® Kit (Qiagen, Toronto, ON, Canada). The 16S ribosomal RNA (rRNA) library preparation and sequencing was performed using the Illumina MiSeq platform at Genome Québec targeting the V3-V4 (Primers: 341F, 805R) region of the 16S rRNA gene.

### Analysis of 16S rRNA sequencing

Forward and reverse, raw, demultiplexed 16S rRNA reads were denoised, chimera filtered, and clustered into sequence variants with Dada2 package (version 1.16) in R (version 4.0.1) [74]. Reads were trimmed at the first instance of a quality score less than or equal to 2 or removed if they contained ambiguous nucleotides (N) or if two or more errors were expected based on the quality of the trimmed read. An average of 14664.19 (±297.35 SEM) high quality 16S rRNA sequences were generated per sample. Amplicon sequence variants (ASVs) were assigned taxonomy using Silva training set v132 [75]. ASVs present in less than 20% of the samples were filtered. Alpha and beta-diversity were computed using the Phyloseq package (version 1.32.0). Statistical significance of weighted UniFrac distance between groups was performed using Adonis function from vegan R package (version 2.5) [76]. Differential abundance analysis of the recovery was performed using metamicrobiomeR package (version 1.30.0) [77]. Metabolic pathway inference was performed by PICRUSt2 software with default parameters (version 2.3.0) [78]. Pathways were then regrouped in functions following BRITE hierarchy and were analyzed with STAMP software (version 2.1.3) [79]. The results of the 16S rRNA profiling are presented in Additional file 1. Graphical representations were performed with ggplot2 R package (version 3.3.2) [80] and GraphPad Prism (version 7.00).

### Real-time PCR

Real time PCR was performed using PowerUp™ SYBR™ Green Master Mix (Thermo Fisher) and the RG 3000A R (Qiagen, Québec, Canada) as described previously [81]. Primers used in the study are presented in Additional file 2, Table 4. Relative quantitation was performed using standard curves constructed from serial dilutions of PCR products [82]. DNA concentration for each targeted sequence was determined by direct comparison with the standard curve of the specific target generated in each PCR run. Expression levels of targeted genes were normalized to 16S rRNA.

### Short chain fatty acids quantification

Quantification of SCFAs was performed using electrospray ionization mass spectrometry (ES-MS) at the Metabolomics Core Facility of the CRCHUM as previously reported [83].

### Statistical analysis

R [84] and STAMP were used to perform statistical analyses. Statistically significant differences were evaluated by two-tailed Welch *t*-test (comparison between two groups or when applicable, paired *t*-test) and when indicated, White’s *t-*test [85]. Multiple testing was corrected using false discovery rate (FDR) [86] estimation.

## Supplementary Information

**Additional file 1.**

**Additional file 2.**

## Data Availability

Sequence data generated and analyzed during the current study are available in the NCBI SRA repository [BioProject PRJNA724731] (https://www.ncbi.nlm.nih.gov/bioproject/PRJNA724731).

## Abbreviations

ASV: Amplicon sequence variants
SCFA: Short-chain fatty acid
ES-MS: Electrospray ionization mass spectrometry
FDR: False discovery rate
PCoA: Principal coordinate analysis
GAMLSS: Generalized additive models for location, scale and shape
BEZI: Beta zero-inflated
Lcn2: Lipocalin 2
NADPH: Nicotinamide adenine dinucleotide phosphate
SD: Standard deviation
SEM: Standard error of the mean
KEGG: Kyoto encyclopedia of genes and genomes
