# Supplementary material for: Oral iron supplementation after antibiotic exposure induces a deleterious recovery of the gut microbiota
Source: BMC Microbiol. 2021 Sep 28;21:259. doi: 10.1186/s12866-021-02320-0 (PMC8480066; doi:10.1186/s12866-021-02320-0)
Supplement: Supplementary file 2 — Additional file 2. [file 12866_2021_2320_MOESM2_ESM.docx]

**Table 1: Changes at the genus level after antibiotic exposure in mice**

| **Phylum** | **Family** | **Genus** | **Estimate** | **95% Lower limit** | **95% Upper limit** | ***p-*value** | ***fdr* adjusted *p*-value** | * |
| --- | --- | --- | --- | --- | --- | --- | --- | --- |
| Bacteroidetes | Bacteroidaceae | *Bacteroides* | -0.17 | -0.24 | -0.1 | <0.0001 | <0.0001 | **↓** |
|  | Rikenellaceae | *Alistipes* | -0.18 | -0.24 | -0.11 | <0.0001 | <0.0001 | **↓** |
|  | Tannerellaceae | *Parabacteroides* | 0.07 | 0.01 | 0.13 | <0.05 | <0.05 | **↑** |
| Firmicutes | Lactobacillaceae | *Lactobacillus* | -0.27 | -0.34 | -0.21 | <0.0001 | <0.0001 | **↓** |
|  | Streptococcaceae | *Lactococcus* | -0.2 | -0.25 | -0.16 | <0.0001 | <0.0001 | **↓** |
|  | Clostridiaceae_1 | *Clostridium_sensu_stricto_1* | -0.52 | -0.59 | -0.46 | <0.0001 | <0.0001 | **↓** |
|  | Lachnospiraceae | *Acetatifactor* | -0.36 | -0.37 | -0.36 | <0.0001 | <0.0001 | **↓** |
|  |  | *GCA_900066575* | -0.62 | -0.65 | -0.6 | <0.0001 | <0.0001 | **↓** |
|  |  | *Lachnoclostridium* | -0.43 | -0.48 | -0.38 | <0.0001 | <0.0001 | **↓** |
|  |  | *Lachnospiraceae_FCS020_group* | -0.47 | -0.47 | -0.46 | <0.0001 | <0.0001 | **↓** |
|  |  | *Lachnospiraceae_NK4A136_group* | -0.35 | -0.4 | -0.29 | <0.0001 | <0.0001 | **↓** |
|  |  | *Lachnospiraceae_UCG_006* | -0.57 | -0.6 | -0.54 | <0.0001 | <0.0001 | **↓** |
|  |  | *Tyzzerella* | -0.32 | -0.37 | -0.27 | <0.0001 | <0.0001 | **↓** |
|  |  | *Tyzzerella_4* | -0.42 | -0.45 | -0.38 | <0.0001 | <0.0001 | **↓** |
|  | Peptostreptococcaceae | *Romboutsia* | -0.6 | -0.61 | -0.6 | <0.0001 | <0.0001 | **↓** |
|  | Ruminococcaceae | *Anaerotruncus* | -0.74 | -0.74 | -0.74 | <0.0001 | <0.0001 | **↓** |
|  |  | *Harryflintia* | -0.29 | -0.35 | -0.24 | <0.0001 | <0.0001 | **↓** |
|  |  | *Intestinimonas* | -0.34 | -0.39 | -0.29 | <0.0001 | <0.0001 | **↓** |
|  |  | *Oscillibacter* | -0.63 | -0.63 | -0.63 | <0.0001 | <0.0001 | **↓** |
|  |  | *Ruminiclostridium* | -0.61 | -0.67 | -0.55 | <0.0001 | <0.0001 | **↓** |
|  |  | *Ruminiclostridium_9* | -0.29 | -0.35 | -0.24 | <0.0001 | <0.0001 | **↓** |
|  |  | *Ruminococcaceae_UCG_004* | 0.32 | 0.25 | 0.38 | <0.0001 | <0.0001 | **↑** |
|  |  | *Ruminococcaceae_UCG_005* | -0.53 | -0.53 | -0.52 | <0.0001 | <0.0001 | **↓** |
|  |  | *UBA1819* | 0.18 | 0.11 | 0.25 | <0.0001 | <0.0001 | **↑** |

***Cont.* Table 1: Changes at the genus level after antibiotic exposure in mice**

| Proteobacteria | Desulfovibrionaceae | *Bilophila* | -0.42 | -0.48 | -0.36 | <0.0001 | <0.0001 | **↓** |
| --- | --- | --- | --- | --- | --- | --- | --- | --- |
|  | Burkholderiaceae | *Parasutterella* | 0.54 | 0.5 | 0.59 | <0.0001 | <0.0001 | **↑** |
| Verrucomicrobia | Akkermansiaceae | *Akkermansia* | 0.18 | 0.12 | 0.24 | <0.0001 | <0.0001 | **↑** |

Longitudinal comparison of relative abundances at the genus level between gut communities in mice before (T0) and after (T7) antibiotic exposure using GAMLSS-BEZI. Only significant changes at the genus level after *fdr* correction are shown. Estimate (regression coefficient) are the log (odds ratio) of relative abundances between comparison groups. *, Arrows indicate an increase (**↑**) or a decrease (**↓**) in relative abundance after antibiotic exposure (T7 vs T0).

**Table 2: Changes at the genus level at the end time point (T80) in mice fed the iron-sufficient diet (50 ppm iron)**

| **Phylum** | **Family** | **Genus** | **Estimate** | **95% Lower limit** | **95% Upper limit** | ***p-*value** | ***fdr* adjusted *p*-value** | * |
| --- | --- | --- | --- | --- | --- | --- | --- | --- |
| Bacteroidetes | Bacteroidaceae | *Bacteroides* | -0.01 | -0.02 | 0 | <0.01 | <0.01 | **↓** |
|  | Rikenellaceae | *Alistipes* | 0.02 | 0.01 | 0.02 | <0.0001 | <0.001 | **↑** |
|  | Tannerellaceae | *Parabacteroides* | 0.01 | 0.01 | 0.02 | <0.01 | <0.01 | **↑** |
| Firmicutes | Lactobacillaceae | *Lactobacillus* | -0.01 | -0.02 | 0 | <0.05 | <0.05 | **↓** |
|  | Streptococcaceae | *Lactococcus* | -0.02 | -0.02 | -0.02 | <0.0001 | <0.0001 | **↓** |
|  | Clostridiaceae_1 | *Clostridium_sensu_stricto_1* | -0.03 | -0.04 | -0.03 | <0.0001 | <0.0001 | **↓** |
|  | Lachnospiraceae | *A2* | -0.02 | -0.02 | -0.01 | <0.0001 | <0.0001 | **↓** |
|  |  | *Acetatifactor* | -0.02 | -0.02 | -0.01 | <0.001 | <0.01 | **↓** |
|  |  | *GCA_900066575* | -0.02 | -0.02 | -0.01 | <0.0001 | <0.0001 | **↓** |
|  |  | *Lachnoclostridium* | -0.01 | -0.01 | -0.01 | <0.0001 | <0.0001 | **↓** |
|  |  | *Lachnospiraceae_FCS020_group* | -0.01 | -0.02 | -0.01 | <0.0001 | <0.001 | **↓** |
|  |  | *Lachnospiraceae_UCG_006* | -0.02 | -0.02 | -0.01 | <0.0001 | <0.0001 | **↓** |
|  |  | *Marvinbryantia* | -0.02 | -0.02 | -0.01 | <0.0001 | <0.0001 | **↓** |
|  |  | *Tyzzerella* | -0.02 | -0.02 | -0.01 | <0.0001 | <0.0001 | **↓** |
|  |  | *Tyzzerella_4* | -0.02 | -0.02 | -0.01 | <0.0001 | <0.0001 | **↓** |
|  | Peptostreptococcaceae | *Romboutsia* | -0.01 | -0.02 | 0 | <0.05 | <0.05 | **↓** |
|  | Ruminococcaceae | *Anaerotruncus* | -0.02 | -0.03 | -0.02 | <0.0001 | <0.0001 | **↓** |
|  |  | *GCA_900066225* | -0.03 | -0.03 | -0.03 | <0.0001 | <0.0001 | **↓** |
|  |  | *Harryflintia* | -0.03 | -0.03 | -0.02 | <0.0001 | <0.0001 | **↓** |
|  |  | *Intestinimonas* | 0 | -0.01 | 0 | <0.05 | <0.05 | **↓** |
|  |  | *Oscillibacter* | -0.02 | -0.02 | -0.01 | <0.0001 | <0.0001 | **↓** |
|  |  | *Ruminiclostridium* | -0.02 | -0.03 | -0.02 | <0.0001 | <0.0001 | **↓** |

***Cont.* Table 2: Changes at the genus level at the end time point (T0) in mice fed the iron-sufficient diet (50 ppm iron)**

|  |  | *Ruminiclostridium_5* | -0.01 | -0.01 | -0.01 | <0.001 | <0.001 | **↓** |
| --- | --- | --- | --- | --- | --- | --- | --- | --- |
|  |  | *Ruminiclostridium_9* | -0.01 | -0.02 | -0.01 | <0.0001 | <0.0001 | **↓** |
|  |  | *Ruminococcaceae_UCG_005* | -0.01 | -0.02 | 0 | <0.05 | <0.05 | **↓** |
|  |  | *Ruminococcaceae_UCG_009* | -0.02 | -0.02 | -0.01 | <0.0001 | <0.0001 | **↓** |
|  |  | *Ruminococcaceae_UCG_014* | -0.02 | -0.02 | -0.01 | <0.001 | <0.001 | **↓** |
|  |  | *UBA1819* | 0.01 | 0.01 | 0.02 | <0.0001 | <0.0001 | **↑** |
| Proteobacteria | Burkholderiaceae | *Parasutterella* | 0.01 | 0 | 0.01 | <0.001 | <0.001 | **↑** |

Longitudinal comparison of relative abundances at the genus level between gut communities in mice at the beginning (T0) and end (T80) of the experiment using GAMLSS-BEZI. Only significant changes at the genus level after *fdr* correction are shown. Estimate (regression coefficient) are the log (odds ratio) of relative abundances between comparison groups. *, Arrows indicate an increase (**↑**) or a decrease (**↓**) in relative abundance during the recovery period compared to the beginning of the experiment (T80 *vs* T0).

**Table 3: Changes at the genus level during the recovery phase after antibiotic treatment in mice fed the iron excess diet (500 ppm iron)**

| **Phylum** | **Family** | **Genus** | **Estimate** | **95% Lower limit** | **95% Upper limit** | ***p-*value** | ***fdr* adjusted *p*-value** | ***** |
| --- | --- | --- | --- | --- | --- | --- | --- | --- |
| Actinobacteria | Eggerthellaceae | *Enterorhabdus* | -0.01 | -0.01 | 0 | <0.01 | <0.01 | **↓** |
| Bacteroidetes | Rikenellaceae | *Alistipes* | 0.01 | 0.01 | 0.02 | <0.01 | <0.01 | **↑** |
|  | Tannerellaceae | *Parabacteroides* | 0.02 | 0.01 | 0.02 | <0.001 | <0.01 | **↑** |
| Firmicutes | Streptococcaceae | *Lactococcus* | -0.02 | -0.02 | -0.01 | <0.0001 | <0.0001 | **↓** |
|  | Clostridiaceae_1 | *Clostridium_sensu_stricto_1* | -0.02 | -0.03 | -0.02 | <0.0001 | <0.0001 | **↓** |
|  | Lachnospiraceae | *A2* | 0.01 | 0 | 0.01 | <0.05 | <0.05 | **↑** |
|  |  | *Acetatifactor* | -0.01 | -0.02 | 0 | <0.01 | <0.05 | **↓** |
|  |  | *GCA_900066575* | -0.02 | -0.02 | -0.01 | <0.0001 | <0.0001 | **↓** |
|  |  | *Lachnoclostridium* | -0.01 | -0.01 | -0.01 | <0.0001 | <0.0001 | **↓** |
|  |  | *Lachnospiraceae_UCG_006* | -0.02 | -0.02 | -0.01 | <0.0001 | <0.0001 | **↓** |
|  |  | *Tyzzerella* | -0.01 | -0.02 | -0.01 | <0.01 | <0.01 | **↓** |
|  |  | *Tyzzerella_4* | -0.02 | -0.02 | -0.01 | <0.0001 | <0.0001 | **↓** |
|  | Peptostreptococcaceae | *Romboutsia* | -0.01 | -0.02 | -0.01 | <0.01 | <0.01 | **↓** |
|  | Ruminococcaceae | *Anaerotruncus* | -0.02 | -0.02 | -0.01 | <0.0001 | 0.0001 | **↓** |
|  |  | *Flavonifractor* | -0.02 | -0.02 | -0.01 | <0.01 | <0.01 | **↓** |
|  |  | *GCA_900066225* | -0.02 | -0.03 | -0.01 | <0.01 | <0.01 | **↓** |
|  |  | *Harryflintia* | -0.03 | -0.03 | -0.02 | <0.0001 | <0.0001 | **↓** |
|  |  | *Intestinimonas* | -0.01 | -0.01 | 0 | <0.01 | <0.01 | **↓** |
|  |  | *Oscillibacter* | -0.01 | -0.02 | -0.01 | <0.0001 | <0.0001 | **↓** |
|  |  | *Ruminiclostridium* | -0.02 | -0.02 | -0.01 | <0.0001 | <0.001 | **↓** |
|  |  | *Ruminiclostridium_5* | -0.02 | -0.03 | -0.02 | <0.0001 | <0.0001 | **↓** |
|  |  | *Ruminiclostridium_9* | -0.01 | -0.02 | -0.01 | <0.001 | <0.01 | **↓** |

***Cont.* Table 3: Changes at the genus level during the recovery phase after antibiotic treatment in mice fed the iron excess diet (500 ppm iron)**

|  |  | *Ruminococcaceae_UCG_005* | -0.01 | -0.02 | 0 | <0.05 | <0.05 | **↓** |
| --- | --- | --- | --- | --- | --- | --- | --- | --- |
|  |  | *Ruminococcaceae_UCG_014* | -0.02 | -0.02 | -0.01 | <0.0001 | <0.0001 | **↓** |
|  |  | *UBA1819* | 0.02 | 0.01 | 0.03 | <0.01 | <0.01 | **↑** |
| Proteobacteria | Desulfovibrionaceae | *Bilophila* | -0.02 | -0.03 | -0.01 | <0.01 | <0.01 | **↓** |
|  | Burkholderiaceae | *Parasutterella* | 0.01 | 0.01 | 0.02 | <0.01 | <0.01 | **↑** |
| Verrucomicrobia | Akkermansiaceae | *Akkermansia* | -0.02 | -0.03 | -0.01 | <0.01 | <0.01 | **↓** |

Longitudinal comparison of relative abundances at the genus level between gut communities in mice at the beginning (T0) and end (T80) of the experiment using GAMLSS-BEZI. Only significant changes at the genus level after *fdr* correction are shown. Estimate (regression coefficient) are the log (odds ratio) of relative abundances between comparison groups. *, Arrows indicate an increase (**↑**) or a decrease (**↓**) in relative abundance during the recovery period compared to the beginning of the experiment (T80 *vs* T0).

**Table 4. Primers**

| **Name** | **Species** | **Sequence** | **Reference** |
| --- | --- | --- | --- |
| BV-1 | *B. vulgatus* | GCATCATGAGTCCGCATGTTC | Bahl M. *et al.* FEMS Microbiology Letters, 2012 |
| BV-2 |  | TCCATACCCGACTTTATTCCTT |  |
| Pexcr-817F | *P. excrementihominis* | AAGTAAAATTCTCAGTAACGCAGC | Frippiat J.P. *et al.* Scientific Reports, 2019 |
| Pexcr-1001R |  | GCTCTCATTACAAGAGCTTCC |  |
| Alistipes f.F | *A. finegoldii* | GTACTAATTCCCCATAACATTCGAG | Tilg *et al.* Cell Host & Microbe, 2016 |
| Alistipes f.R |  | CTAATACAACGCATGCCCATCTT |  |

**Additional figure 1.**


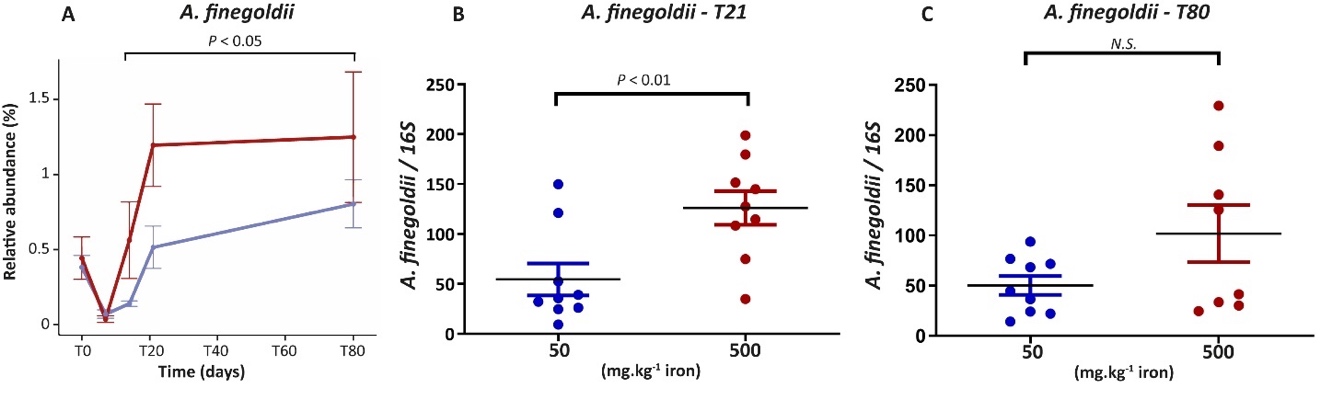


**Relative abundance of bacterial species in iron-supplemented mice after antibiotic exposure.** (A) Relative abundance of *A. finegoldii* detected as significantly different by GAMLSS-BEZI in mice fed an iron excess diet compared to mice fed an iron-sufficient diet during the recovery phase. (N = 9 per time point and group). (B-C) Real-time PCR analysis of the abundance of *A. finegoldii* at (B) T21 and (C) T80 normalized to 16S rRNA gene (N = 9 per time group).
